# Pelvic Floor Rehabilitation After Rectal Cancer Surgery: One-year Follow-up of a Multicenter Randomized Clinical Trial (FORCE Trial)

**DOI:** 10.1097/SLA.0000000000006402

**Published:** 2024-06-20

**Authors:** Noes Margaretha Bosch, Jenneke Aaltje Kalkdijk-Dijkstra, Hendrik Leendert van Westreenen, Paul ma Broens, Jean Pierie Eugène Nicolas, Joost Albertus Gerardus van der Heijden, Bastiaan Rijk Klarenbeek

**Affiliations:** * Department of Surgery, Radboud University Medical Center, Nijmegen, the Netherlands; † Department of Surgery, University Medical Centre Groningen, Groningen, the Netherlands; ‡ Department of Surgery, Isala Hospitals, Dokter Van Heesweg 2, Zwolle, The Netherland; § Department of Surgery, Isala Clinics, Zwolle, the Netherlands; ∥ Department of Surgery, Medical Center Leeuwarden, Leeuwarden, the Netherlands

**Keywords:** fecal incontinence, functional outcomes, low anterior resection syndrome, low anterior resection, pelvic floor rehabilitation, quality of life, rectal cancer

## Abstract

**Objective:**

To evaluate the effects of pelvic floor rehabilitation (PFR) after low anterior resection (LAR) at 1-year follow-up.

**Background:**

After LAR, with restoration of bowel continuity, up to 90% of patients develop anorectal dysfunction, significantly impacting their quality of life (QoL). However, standardized treatment is currently unavailable. The FORCE trial demonstrated the beneficial effects of PFR after 3 months regarding specific domains of the Fecal Incontinence QoL (FIQL) questionnaire and urgency compared with usual care.

**Methods:**

The FORCE trial is a multicenter, two-arm, randomized clinical trial. All patients undergoing LAR were randomly assigned to receive either usual care or a standardized PFR program. The primary outcome measure is the Wexner incontinence score, and the secondary endpoints included the low anterior resection syndrome score, the European Organization for Research and Treatment of Cancer colorectal-specific QoL questionnaire, and health and FIQL. Assessments were conducted at baseline before randomization, at 3 months and 1-year follow-ups.

**Results:**

A total of 86 patients were included (PFR: n = 40, control: n = 46). After 1 year, PFR did not significantly improve Wexner incontinence scores (PFR: -3.33, 95% CI: -4.41 to -2.26, control: -2.54, 95% CI: -3.54 to -1.54, *P* = 0.30). Similar to the 3-month follow-up, patients without near-complete incontinence at baseline showed a sustained improvement in fecal incontinence (PFR: -2.82, 95% CI: -3.86 to -1.76, control: -1.43, 95% CI: -2.36 to -0.50, *P* = 0.06). Significant improvement was reported in the FIQL domains Lifestyle (PFR: 0.51, control: -0.13, *P* = 0.03) and Coping and Behavior (PFR: 0.40, control: -0.24, *P* = 0.01).

**Trial registration:**

Netherlands Trial Registration, NTR5469, registered on September 3, 2015.

**Conclusions:**

At 1-year follow-up, no significant differences were found in fecal incontinence scores; however, PFR was associated with improved FIQL compared with usual care.

After a low anterior resection (LAR), up to 90% of patients experience a collection of symptoms known as low anterior resection syndrome (LARS), which can have a significant impact on physical health and quality of life (QoL).^1–6^ Fecal incontinence (FI), in particular, greatly affects QoL.^4–8^ One promising therapy for LARS is pelvic floor rehabilitation (PFR), currently used for nonsurgical patients with FI.^9,10^

The FORCE trial investigated the effects of PFR in a postsurgery population, specifically after LAR for rectal cancer, using the Wexner incontinence score and the Fecal Incontinence QoL (FIQL) as primary outcomes. The trial reported no significant improvement in Wexner incontinence scores after 3 months of PFR in nonselected patients.^7^ However, a significant improvement in Wexner incontinence score after PFR was found for patients without near-complete incontinence, accounting for ∼80% of the total group. Furthermore, significant improvements in all domains of the FIQL scale were observed in subsets of patients, specifically those with at least moderate incontinence.^7^ These findings suggest the potential for a selective referral policy for postoperative PFR. This long-term follow-up study is crucial to investigate the sustainability or divergence of the effects observed at 3 months, as the effects on QoL after prolonged follow-up remain uncertain. Guillemot et al^11^ demonstrated a more significant improvement in incontinence scores at 6 months compared with 30 months, indicating a decrease in effects over time.

In addition, approximately half of the patients who undergo LAR continue to struggle to experience LARS symptoms throughout their lives.^12,13^ Given the prevalence of patients with rectal cancer, improved survival, and the persistent nature of LARS symptoms, it is important to investigate whether the suggested selective referral policy based on the 3-month results is sustainable. The hypothesis is to find a sustained effect over time, as several previous studies have shown sustained effects after a prolonged follow-up period.^14–17^

## METHODS

### Study Design and Population

**FIGURE 1:**
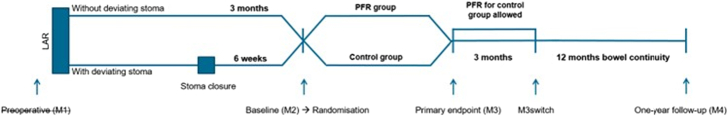
Study overview. M1 indicates preoperative measurement (crossed out because it has been removed from the study protocol); M2, baseline measurement; M3, primary endpoint; M4, 1-year follow-up.

This study presents the 1-year follow-up data of the FORCE trial, a multicenter, two-armed, randomized controlled trial that investigated the effects of PFR on the severity of FI and QoL. To ensure the feasibility of the study, a preoperative measurement (M1) was removed from the study protocol as it hampered inclusion rates.^18^ Baseline assessments were conducted before randomization (M2), which occurred either 3 months after LAR without a stoma or 6 weeks after stoma closure. The primary results were assessed 3 months after the start of the intervention (M3). The final assessments took place 1 year after surgery or repair of bowel continuity (in the case of a temporary stoma; M4). A small group of participants (n = 5) who were initially assigned to the control group later opted for the intervention after completion of the regular M3 assessments. They are referred to as “M3switch” (Fig. 1). The follow-up period of 1 year was chosen based on the observed spontaneous recovery of bowel function during the first 6 to 12 months after sphincter-preserving surgery, as reported by van Duijvendijk et al.^19^

The standard care includes pharmacotherapy such as bulking agents and/or antidiarrheal medication, disclosed to both study arms. Patients in either group were allowed to use these medications and were asked to log their usage.^18^ In the control group, both the oncological and functional follow-up was provided by their own medical professionals in accordance with the guidelines. Treatment of patients in the standardized intervention group consists of 4 modalities, besides standard treatment: (1) pelvic floor muscle training to enhance strength, duration, and coordination, (2) Biofeedback using an anal electromyography probe, (3) electrostimulation to improve muscle contraction, and (4) training with a rectal balloon to simulate urgency and enhance stool retention. Patients participated individually in weekly treatment sessions for 3 months. A detailed description of PFR was published previously.^18^ The PFR physiotherapists underwent training with the aim of structuring the sessions and ensuring proper execution. Moreover, they maintained a detailed case report for each session. The use of medication was reported in the case report form too.

The study population consisted of patients with rectal cancer who underwent LAR. In each participating center, a principal investigator was responsible for participant inclusion and obtaining informed consent. The inclusion criteria were as follows: (1) adult aged ≥18 years, (2) LAR for rectal carcinoma, (3) intellectual and linguistic capability to understand the questionnaires in Dutch, and (4) completion of a 1-year follow-up. Patients with a medical history of Crohn disease, ulcerative colitis, or proctitis, those who were mentally or physically unable to undergo PFR, and those with a life expectancy of <1 year were excluded from the trial. Reasons for not completing a 1-year follow-up were recorded. Subgroup analyses were performed as described in the original FORCE trial publication, including patients with at least moderate incontinence at baseline (Wexner incontinence score ≥5), those without near-complete incontinence at baseline (Wexner incontinence ≤16), and a combination of at least moderate incontinence at baseline and no near-complete incontinence.^7^

### Outcomes

The primary outcome measure was the validated Wexner incontinence score, which ranged from 0 to 20. Scores equal to or higher than 1 were considered symptomatic; with mild incontinence defined as scores between 1 and 4, moderate incontinence between 5 and 8, and severe incontinence between 9 and 20.^20–22^ A clinically relevant change was defined as a minimum difference of two points.^22^ Wexner scores equal to or higher than 16 were classified as near-complete incontinence^7,23,24^ (Table 1; Wexner FI Score).^25^

**TABLE 1:** Wexner FI Score^25^

	Frequency				
Type of incontinence	Never	Rarely	Sometimes	Usually	Always
Solid	0	1	2	3	4
Liquid	0	1	2	3	4
Gas	0	1	2	3	4
Wears pad	0	1	2	3	4
Lifestyle alteration	0	1	2	3	4

Never = 0; rarely = <1/months; sometimes = <1week, ≥1/month; usually = <1/day, ≥1/week, always = ≥1/day.

0 = perfect; 20 = complete incontinence.

The secondary outcome measures included the FIQL Scale, which ranged from 1 to 4 (poor to good QoL), with a minimal clinically important difference of 0.4 points.^26^ The LARS score, ranging from 0 to 42, categorized the presence of no, minor, or major LARS. In addition, the European Organization for Research and Treatment of Cancer colorectal specific QoL questionnaire (EORTC QLQ-CR29) assessed various functional and symptom scales, with scores ranging from 0 to 100. Higher function scores indicated better outcomes, whereas higher symptom scores indicated more complaints. A minimal clinically relevant change was defined as a difference of at least 5 points between baseline and follow-up, as well as a difference of at least 5 points compared with the competing study arm.^27^ The symptom domains included in the LARS score were also evaluated individually, including flatus (occurring at least once a week), liquid stool (occurring at least once a week), frequency (more than seven times per day), clustering (occurring at least once a week), and urgency (occurring at least once a week).

### Data Analysis and Statistics

A power analysis was made up-front with an analysis of covariance (ANCOVA) as the primary statistical test; details are provided in the study protocol for this trial.^18^ The mean change in Wexner incontinence score was compared at different time points (M2, M3, and M4) and between groups using ANCOVA. Similar to the analysis of the 3-month results,^7^ regression analysis was employed to identify factors significantly influencing the baseline Wexner score, which was then included as covariates in the analysis. The identified factors included age, preoperative tumor height, and neoadjuvant treatment. As not all differences at baseline could be explained by these factors, the baseline Wexner score was also included as a covariate. The secondary outcome measures (FIQL, LARS, EORTC QLQ CR29) were also analyzed using the ANCOVA test, with their respective baseline scores as covariates. Categorical variables were compared using the χ^2^ test or Fisher exact test. The effect of the intervention and control group was evaluated both with and without the “M3switch” group to assess the influence of this group on long-term data. The primary analysis was based on the intention-to-treat principle. In addition, a per-protocol analysis was performed. A sensitivity analysis was conducted to handle missing data (patients who completed 3 months but failed the 1-year follow-up) using different imputation methods, including no imputation, optimistic imputation (the best possible score), pessimistic imputation (the worst possible score), and mean imputation of results. Ultimately, missing data were treated as missing, as no statistically or clinically significant differences were found in the sensitivity analyses mentioned above. Two-sided *P* values or 95% CIs were reported, with *P* values <0·05 considered significant. The data were analyzed using IBM SPSS version 25·0 (IBM).

## RESULTS

From October 2017 to March 2020, a total of 106 patients were included and randomly assigned to either the PFR group (51 patients) or the usual care group (55 patients). Seven patients did not reach the 3-month follow-up, and an additional 13 patients did not reach the 1-year follow-up. Among these, 8 patients from the PFR group and 5 patients from the control group did not complete the 1-year follow-up due to personal reasons (1 in the PFR group, 2 in the control group), non-oncology comorbidity (3 in the PFR group, 1 in the control group), and recurrence of cancer (4 in the PFR group, 2 in the control group). Therefore, a total of 86 patients were included in the analysis at M4, with 40 in the PFR group and 46 in the control group. Ten patients were excluded from the per-protocol population, including 8 in the PFR group (received other treatment than randomization; n = 3, incapability to complete PFR; n = 5) and 2 in the control group (randomized into the control group but independently opted to participate in PFR; Table 2, for the patient characteristics, and Supplemental File 1, Supplemental Digital Content 1, http://links.lww.com/SLA/F157, for the additional information on the per-protocol population).

**TABLE 2:** Patient Characteristics

	Intention to treat		
	Control group (n = 46)*	PFR group (n = 40)*	*P*
Demographics			
Age (yr), median (IQR)	63.0 (17.0)	63.0 (11.0)	0.62
Sex (M), n (%)	30 (65.2)	23 (57.5)	0.47
Body mass index (kg/m^2^), median (IQR)	26.0 (5.3)	24.90 (6.5)	0.80
Medical history			
Diabetes mellitus, n (%)	4 (8.7)	5 (12.5)	0.57
History of anal surgery, n (%)†	7 (15.2)	5 (12.5)	0.72
ASA classification, n (%)			0.96
ASA 1-2	42 (91.3)	32 (80.0)	—
ASA 3-4-5	4 (8.7)	8 (20.0)	—
Tumor characteristics			
Tumor height in cm (MRI), mean (SD)	8.8 (3.5)	7.8 (3.7)	0.13
Anastomosis height in cm from anal verge, mean (SD)	5.9 (2.9)	6.0 (2.4)	0.90
(y-) Pathologic TNM stage, n (%)			
T0	2 (4.3)	0	0.87
T1	8 (17.4)	3 (7.5)	—
T2	13 (28.3)	20 (50.0)	—
T3	21 (45.7)	17 (42.5)	—
T4	2 (4.3)	0	—
N0	28 (60.9)	33 (82.5)	0.86
N1	17 (37.0	7 (17.5)	—
N2	1 (2.2)	0	—
M0	41 (89.1)	40 (100.0)	0.86
M1‡	2 (4.3)	0	—
Mx	3 (6.5)	0	—
Additional therapy			
Neoadjuvant therapy, n (%)	22 (47.8)	18 (45.0)	0.80
Radiotherapy short course (5×5 Gy)	10 (45.5)	7 (38.9)	—
Chemoradiation	12 (54.5)	11 (61.1)	—
Adjuvant chemotherapy, n (%)	1 (2.2)	1 (2.5)	0.92
Surgery related factor			
Type of surgery, n (%)			0.57
Laparoscopic	38 (82.6)	31 (77.5)	—
Robot	7 (15.2)	8 (20.0)	—
Conversion to open	1 (2.2)	1 (2.5)	—
Construction of a stoma, n (%)§	25 (54.3)	23 (57.5)	0.43
No stoma construction	21 (45.7)	17 (42.5)	—
Yes, stoma construction	19 (90.5)	17 (100.0)	—
Ileostomy	2 (9.5)	0	—
Colostomy	—	—	—
Time to stoma closure (mo), median (IQR)	3.0 (4.0)	3.0 (2.0)	0.59
Stoma closure, n (%)	21 (54.3)	17 (42.5)	0.63
Within 3 mo	7 (33.30)	7 (41.20)	—
After 3 mo	14 (66.70)	10 (58.80)	—
Type of anastomosis, n (%)	37 (80.4)	29 (70.0)	0.36
Side-to-end	9 (19.6)	11 (27.5)	—
End-to-end	0	1 (2.5)	—
Not reported adequately			
Anastomotic leakage, n (%)	1 (2.2)	3 (7.5)	0.25
Obstetric history, n = females	16	17	—
No. of women who delivered a child, n (%)	14 (87.5)	13 (76.0)	0.81
Woman with only vaginal deliveries, n (%)	12 (92.3)	13 (100.0)	0.33
Presence of vaginal tears or episiotomy procedures, n (%)	8 (66.7)	5 (38.5)	0.17

* Number of patients that completed measurements at M4.

† Control group: a history of surgeryfor fistula (1x), endoscopic mucosal resection (EMR, 1x), endoscopic submucosal dissection (ESD, 1x), Transanal Endoscopic Microsurgery (TEM)/wait and see (3x). PFR group: a history of surgery for fistula (1x), TEM (1x), unknown type of surgery 20 years ago (1x).

‡ All synchronic metastasis that were treated by primary resection (1x, control group), ablation (1x, control group) or complete response after neoadjuvant treatment (1x, control group), all before LAR.

§ At index surgery or constructed in the first postoperative period (i.e. due to complications).

ASA indicates American Society of Anaesthesiologists; IQR, interquartile range; MRI, Magnetic Resonance Imaging.

No significant differences were found in patient characteristics. In the PFR group, 58.1% of the patients were men, while in the control group, it was 64.0%. The median age at inclusion was 63.0 years for both groups (interquartile range: 11 for the PFR and 17 for the control group). The mean preoperative tumor height was 7.1 cm (SD: 2.9) in the PFR group and 8.4 cm (SD: 3.3) in the control group. Neoadjuvant therapy consisted of 33.3% radiotherapy and 66.7% chemoradiation in the PFR group, and 45.8% radiotherapy and 54.2% chemoradiation in the control group. A temporary stoma had been constructed in 46.5% of the PFR group and 44.0% of the control group, with a closure within 3 months. In addition, 77.8% of the woman in the PFR group and 72.2% in the control group had given birth through vaginal delivery.

### Primary Outcome Measure

**FIGURE 2:**
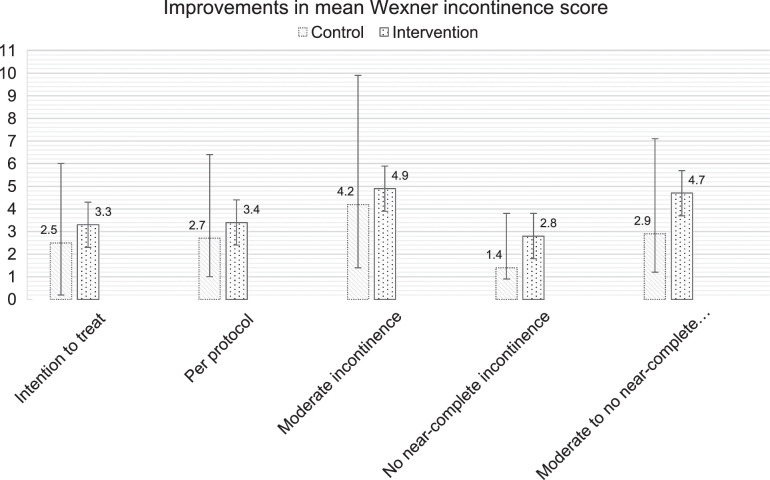
Improvement in mean Wexner incontinence score per subgroup. 95% CIs are shown. At least moderate incontinence (baseline Wexner score ≥5), no near-complete incontinence (baseline Wexner score <16), and at least moderate to no near-complete incontinence (baseline Wexner score ≥5 and <16).

No significant difference in Wexner incontinence scores was found between the PFR and control groups when comparing baseline and 1-year follow-up (PFR: -3.33, 95% CI: -4.41 to -2.26 versus control: -2.54, 95% CI: -3.54 to -1.54, *P* = 0.30) after adjusting for age, preoperative tumor height, neoadjuvant treatment, and baseline Wexner score. In the per-protocol population, the adjusted difference in the PFR group was -3.35 (95% CI: -4.50 to -2.20), and in the control group, it was -2.67 (95% CI: -3.66 to -1.68, *P* = 0.39). Sensitivity analyses did not reveal any differences in the results. There were improvements in Wexner incontinence scores after PFR in patients without near-complete incontinence at baseline, although not significant (PFR: -2.81, 95% CI: -3.86 to -1.76, n = 32, control: -1.43, 95% CI: -2.36 to -0.50, n = 40, *P* = 0.06). This finding is consistent with the sustained effect observed in the 3-month follow-up data (PFR: –2.1, n = 36, control: –0.7, n = 45, *P* = 0.045). However, no improvement in incontinence scores after PFR was observed in patients with at least moderate incontinence at baseline (PFR: -4.93, n = 32, control: -4.23, n = 27, *P* = 0.49). Evidence was found of improvement in patients between moderate and near-complete incontinence at baseline, although not significant (PFR: -4.74, n = 24, control: -2.96, n = 21, *P* = 0.07). There were no significant changes in scores from the 3-month follow-up to the 1-year follow-up (Fig. 2 and Table 3 for the Wexner incontinence scores, and Supplemental File 2, Supplemental Digital Content 1, http://links.lww.com/SLA/F157 for the per-protocol population analyses and differences between 3 months and 1-year follow-ups).

**TABLE 3:** Primary Outcome Wexner Incontinence Score

		Intention to Treat		
Outcomes	Timing of measurement	Control group (n = 46)^*^	Intervention group (n = 40)^*^	*P*
Total Wexner score, median (IQR)	M2	6.00 (12.00)	11.50 (8.00)	—
	M3	5.00 (7.00)	6.00 (10.00)	—
	M4	4.50 (7.00)	5.00 (6.00)	—
Change scores (M4-M2)	Adjusted mean difference ^†^	-2.54 95% CI: -3.54 to -1.54	-3.33 95% CI: -4.41 to -2.26	0.30
Change scores (M4-M3),	Adjusted mean difference ^†^	-1.02 95% CI: -1.99 to -0.05	-1.02 95% CI: -2.07 to 0.02	0.999
At least moderate incontinence		n = 27^*^	n = 32^*^	*P*
Total Wexner score, median (IQR)	M2	12.00 (9.00)	12.50 (7.00)	—
	M3	6.00 (8.00)	7.50 (8.00)	—
	M4	7.00 (7.00)	6.50 (6.75)	—
Change scores (M4-M2)	Adjusted mean difference ^†^	-4.23 95% CI: -5.69 to -2.77	-4.93 95% CI: -6.27 to -3.59	0.49
Change scores (M4-M3)	Adjusted mean difference ^†^	-1.40 95% CI: -2.81 to 0.02	-1.42 95% CI: -2.71 to -0.12	0.98
No near-complete incontinence		n = 40^*^	n = 32^*^	*P*
Total Wexner score, median (IQR)	M2	5.00 (9.00)	9.50 (8.00)	—
	M3	5.00 (4.00)	5.00 (6.00)	—
	M4	4.00 (6.50)	4.00 (5.50)	—
Change scores (M4-M2)	Adjusted mean difference ^†^	-1.43 95% CI: -2.36 to -0.50	-2.81 95% CI: -3.86 to -1.76	0.06
Change scores (M4-M3)	Adjusted mean difference ^†^	-0.55 95% CI: -1.45 to .34	-0.75 95% CI: -1.76 to 0.27	0.79
Between moderate and near-complete incontinence		n = 21	n = 24	*P*
Total Wexner score, median (IQR)	M2	11.00 (8)	11.50 (6)	—
	M3	6.00 (6)	6.50 (6)	—
	M4	6.00 (5.5)	5.50 (5.75)	—
Change scores (M4-M2), mean (95% CI)	Unadjusted mean difference	-2.90 (-4.22 till -1.57)	-4.51 (-5.75 till -3.27	0.083
	Adjusted mean difference ^†^	-2.96 (-4.22 till -1.70)	-4.74 (-6.03 till -3.45)	0.065
Change scores (M4-M3), mean (95% CI)	Unadjusted mean difference	-0.70 (-2.03 till 0.63)	-1.10 (-2.34 till 0.14)	0.662
	Adjusted mean difference ^†^	-0.76 (-2.06 till 0.53)	-0.85 (-2.18 till 0.48)	0.930

M2: baseline measurement. M3: primary endpoint. M4: 1-year follow-up.

Change scores are displayed in mean (95% CI).

^*^ Number of participants who completed the M4 measurements.

^†^ Analysis of covariance with mean change in Wexner incontinence score (M2-M3 and M3-M4) adjusted for age, preoperatively assessed tumor height from anal verge, neoadjuvant treatment, and Wexner baseline score.

IQR indicates interquartile range.

### Secondary Outcome Measures Fecal Incontinence-related Quality of Life After One Year

Regarding FIQL after 1 year, improvements were observed in nonselected patients who underwent PFR compared with baseline assessments. These patients demonstrated better scores in the FIQL domains Lifestyle (PFR: 0.51, control: -0.13, *P* = 0.03) and Coping and Behavior (PFR: 0.40, control: -0.24, *P* = 0.01).

In patients with at least moderate incontinence at baseline, there was improvement at the 1-year follow-up in the FIQL domains of Lifestyle (PFR: 0.73, n = 32; control: 0.14, n = 27, *P* = 0.048), Coping and Behavior (PFR: 0.64; control: 0.01, *P* = 0.01), and Depression and Self-Perception (PFR: 0.26; control: -0.27, *P* = 0.049). Similarly, patients without near-complete incontinence at baseline showed improved FIQL scores at 1-year follow-up after PFR in the domains Lifestyle (PFR: 0.51; control: -0.26, *P* = 0.03) and Coping and Behavior (PFR: 0.43; control: -0.32, *P* < 0.01). In addition, the group with at least moderate and no near-complete incontinence at baseline reported statistically significant improvement in the domains of Lifestyle (PFR: 0.79; control: 0.02, *P* = 0.03) and Coping and Behavior (PFR: 0.73; control: -0.04, *P* < 0.01). However, no significant differences were found in the FIQL domains of Depression and Self-Perception and Embarrassment for all the mentioned groups (Supplemental File 3, Supplemental Digital Content 1, http://links.lww.com/SLA/F157 for the secondary outcome results).

EORTQ QoL scores and LARS score/categories There were no statistically significant differences in the LARS and EORTC QLQ-CR29 scores between baseline and 1-year follow-up assessments (Supplemental File 3, Supplemental Digital Content 1, http://links.lww.com/SLA/F157 for a comprehensive overview of the results).

At baseline, urgency was reported in 29.4% (n = 15) of the patients in the control group and 61.4% (n = 27) in the PFR group. After 3 months, 25.5% (n = 13) of the patients in the control group and 25.0% (n = 11) in the PFR group reported urgency. A multivariable logistic regression analysis, accounting for urgency and preoperative tumor height at baseline, did not show a significant association between PFR and urgency at 1-year follow-up (odds ratio: 1.36, *P* = 0.63). In addition, no significant associations were found between PFR and the presence of stool frequency, flatus, clustering, and liquid stool.

## DISCUSSION

This long-term follow-up study of the randomized controlled FORCE trial aimed to assess the sustained effects of PFR on functional outcomes and QoL 1 year after restored bowel continuity after LAR for rectal cancer. The results of the QoL data and incontinence Wexner score indicate that the improvements observed after 3 months are sustainable, supporting the recommendation for a selective referral policy. In the 3-month follow-up trial, PFR demonstrated improvement in FI scores compared with usual care for patients without near-complete incontinence.^7^ This effect was also observed in the 1-year follow-up analysis, although not significant. There are several potential explanations for this finding. Firstly, it is likely that any differences between the groups at longer follow-up periods are diminished due to natural recovery over time in the control group. Secondly, it is possible that patients did not continue to engage in pelvic floor muscle exercises after the intervention ended, resulting in the loss of strength and coordination gained during the treatment. This phenomenon has been observed in a 5-year follow-up study on pelvic floor muscle training for stress urinary incontinence.^28^ However, there have also been studies where the effects of PFR were sustained over time. Heymen et al^29^ investigated the effects of manometric biofeedback-guided rectal balloon training on FI and reported a reduction in severity and frequency that persisted at a 1-year follow-up.

An essential aspect of the trial involves coaching patients during the PFR sessions. This allowed participants to engage with health care professionals, enabling them to receive more guidance and specific answers to their questions. This personalized approach not only aids patients with valuable information but also enhances their ability to comprehend and manage their complaints effectively. The control arm did not receive this guidance, and this might contribute to the differences found. However, due to the multifaceted nature of LARS, the QoL and physical improvement cannot be discerned. Although no statistically significant differences in FI status were found, the study observed improvements in FIQL at 3 months of follow-up that were sustained at the 1-year follow-up. These improvements were observed in nonselected patients, as well as in patients with at least moderate incontinence or no near-complete incontinence. In contrast, the control group mostly showed deterioration in FIQL. These findings regarding FIQL align with other literature, such as the study by Nishigori et al.^30^ Their study reported significantly improved outcomes in all 4 FIQL domains after 6 months of muscle training and biofeedback in patients after LAR. However, they also found a significant improvement in Wexner incontinence score after 6 months of PFR, which was not observed in the present study at the 12-month mark. This difference in follow-up duration may partially explain the disparity in incontinence scores, as spontaneous recovery of bowel function is known to continue until 12 months.^19^ In addition, the absence of a control group in the study by Nishigori et al,^30^ which was the case in many previous studies showing a positive effect of PFR on FI,^31^ could contribute to the differing outcomes. The present study’s findings are similar to those of Laforest et al,^32^ where a significant improvement in FIQL domains was found after PFR after rectal cancer surgery, despite no significant improvement in the Wexner score. A noteworthy finding in this study relates to the FIQL embarrassment score. Both the PFR and control groups scored worse at the 1-year follow-up compared with their baseline scores (PFR: -0.09, control: -0.58, *P* = 0.11). However, the control group experienced a clinically significant deterioration, whereas patients who underwent PFR did not. There is no clear explanation for this observation in the literature. It is conceivable that a small reduction in complaints immediately after surgery may initially feel positive. However, over a longer period, patients may not perceive any improvement and may perceive their level of functioning as it was before, which could contribute to their feelings of embarrassment. Another possible factor could be that patients score worse on embarrassment later on due to their inability to achieve the desired results despite their efforts in PFR to reduce their complaints. The main limitations of this follow-up study are similar to the 3-month follow-up study, namely the presence of substantial differences in baseline Wexner scores despite adequate randomization using a computerized sequence generator and appropriate allocation to the study arms.^7^ This is likely due to the study being underpowered.^18^

Given that the FORCE trial was the first randomized comparative trial evaluating PFR, this follow-up study is the first of its kind to examine the long-term effects. A strength of this study is that the participating pelvic floor physiotherapists were trained to adhere to the FORCE trial protocol, which included 4 relevant training modalities.^18,33–35^ Moreover, the questionnaires utilized in this study, such as the Wexner incontinence score and FIQL score, are considered to provide more objective outcomes^22^ and offer better insights into the QoL after LAR, including a specific colorectal module (EORTC QLQ-CR29).^36^ In addition, the inclusion of patients, regardless of the degree of their complaints, allows for a broader statement on the efficacy of PFR in all patients and subgroups, facilitating the design of future studies. In our opinion, future research should focus on identifying predictive factors for the therapeutic success of PFR, utilizing the subgroup analyses provided in this study for this purpose. The current study results have practical implications in guiding the referral of postoperative patients for PFR. Based on the findings, it is recommended to refer patients who do not have near-complete incontinence, indicated by a Wexner score below 16, for PFR.

## CONCLUSIONS

Similar to the results observed after 3 months, this study demonstrates that patients without near-complete incontinence after LAR for rectal cancer show improvements in the Wexner incontinence score and QoL. These improvements are sustained over time, providing further support for the implementation of a selective referral policy for PFR following this type of surgery.

## Supplementary Material
