# Supplementary material for: Pelvic Floor Rehabilitation After Rectal Cancer Surgery: One-year Follow-up of a Multicenter Randomized Clinical Trial (FORCE Trial)
Source: Ann Surg. 2024 Jun 20;281(2):235–42. doi: 10.1097/SLA.0000000000006402 (PMC11723484; doi:10.1097/SLA.0000000000006402)
Supplement: Supplementary file 1 [file sla-281-235-s001.docx]

**Supplementary file 1. Patient characteristics of the per protocol population.**Abbreviations: IQR, interquartile range. ASA-classification, The American Society of Anaesthesiologists (ASA) physical status classification system. TNM, tumour-node-metastasis classification system.

|  | | | | | | Intention to treat | | | | | | Per protocol | | | | | |  |  |  |  |
| --- | --- | --- | --- | --- | --- | --- | --- | --- | --- | --- | --- | --- | --- | --- | --- | --- | --- | --- | --- | --- | --- |
|  |  |  |  |  |  | **Control group  n=46**** | | **PFR group n=40**** | **P-value** | | | **Control group  n=44**** | | **PFR group  n=33**** | | **P-value** | |  |  |  |  |
|  | |  | | Demographics | | | | | | | | | | | | | | | | | |
| Age, years, median (IQR) | | | | | | 63.0 (17.0) | | 63.0 (11.0) | | | 0.62 | 63.0 (18.0) | | 63.0 (13.0) | | 0.84 | |  |  |  |  |
| Males, n (%) | | | | | | 30 (65.2) | | 23 (57.5) | | | 0.47 | 28 (63.6) | | 19 (57.6) | | 0.60 | |  |  |  |  |
| Body mass index, kg/m^2^, median (IQR) | | | | | | 26.0 (5.3) | | 24.90 (6.5) | | | 0.80 | 26.0 (5.7) | | 24.9 (6.6) | | 0.74 | |  |  |  |  |
|  | |  | | Medical history | | | | | | | | | | | | | | | | | |
| Diabetes mellitus, n (%) | | | | | | 4 (8.7) | | 5 (12.5) | 0.57 | | | 4 (9.1) | | 3 (9.1) | | 1.00 | |  |  |  |  |
| History of anal surgery, n (%) ^1^ | | | | | | 7 (15.2) | | 5 (12.5) | 0.72 | | | 6 (13.6) | | 3 (9.1) | | 0.55 | |  |  |  |  |
| ASA classification, n (%)  ASA 1-2  ASA 3-4-5 | | | | | | 42 (91.3)  4 (8.7) | | 32 (80.0)  8 (20.0) | 0.96 | | | 41 (93.2)  3 (6.8) | | 27 (81.8)  6 (18.2) | | 0.26 | |  |  |  |  |
|  | |  | | Tumour characteristics | | | | | | | | | | | | | | | | | |
| Tumour height in cm (MRI), mean (SD) | | | | | | 8.8 (3.5) | | 7.8 (3.7) | 0.13 | | | 9.1 (3.4) | | 7.9 (3.9) | | 0.67 | |  |  |  |  |
| Anastomosis height in cm from anal verge, mean (SD) | | | | | | 5.9 (2.9) | | 6.0 (2.4) | 0.90 | | | 5.9 (3.0) | | 6.0 (2.4) | | 0.98 | |  |  |  |  |
| (y-) Pathological TNM stage, n (%)  T0  T1  T2  T3  T4  N0  N1  N2  M0  M1 ^2^  Mx | | | | | | 2 (4.3)  8 (17.4)  13 (28.3)  21 (45.7)  2 (4.3)  28 (60.9)  17 (37.0  1 (2.2)  41 (89.1)  2 (4.3)  3 (6.5) | | 0 (0.0)  3 (7.5)  20 (50.0)  17 (42.5)  0 (0.0)  33 (82.5)  7 (17.5)  0 (0.0)  40 (100.0)  0 (0.0)  0 (0.0) | 0.87  0.86  0.86 | | | 2 (4.5)  8 (18.2)  12 (27.3)  20 (45.5)  2 (4.5)  27 (61.4)  16 (36.4)  1 (2.3)  39 (88.6)  2 (4.5)  3 (6.8) | | 0 (0.0)  3 (9.1)  17 (51.5)  13 (39.4)  0 (0.0)  26 (78.8)  7 (21.2)  0 (0.0)  33 (100.0)  0 (0.0)  0 (.000) | | 0.83  0.84  0.85 | |  |  |  |  |
|  | |  | | Additional therapy | | | | | | | | | | | | | | | |  |  |
| Neoadjuvant therapy, n (%)  Radiotherapy short course (5x5 Gy)  Chemoradiation | | | | | | 22 (47.8) 10 (45.5) 12 (54.5) | | 18 (45.0) 7 (38.9) 11 (61.1) | 0.80 | | | 20 (45.5) 8 (40.0) 12 (60.0) | | 16 (48.5) 7 (43.8) 9 (56.3) | | 0.80 | |  |  |  |  |
| Adjuvant chemotherapy, n (%) | | | | | | 1 (2.2) | | 1 (2.5) | 0.92 | | | 1 (2.3) | | 1 (3.0) | | 0.84 | |  |  |  |  |
|  | |  | | Surgery related factor | | | | | | | | | | | | | | | | | |
| Type of surgery, n (%)  Laparoscopic  Robot  Conversion to open | | | | | | 38 (82.6)  7 (15.2)  1 (2.2) | | 31 (77.5)  8 (20.0)  1 (2.5) | 0.57 | | | 37(84.1)  6 (13.6)  1 (2.3) | | 25 (75.8)  7 (21.2)  1 (3.0) | | 0.39 | |  |  |  |  |
| Construction of a stoma, n (%) ^3^  No stoma construction  Yes, stoma construction  Ileostomy  Colostomy | | | | | | 25 (54.3)  21 (45.7) 19 (90.5)  2 (9.5) | | 23 (57.5)  17 (42.5)  17 (100.0)  0 (0.0) | 0.43 | | | 24 (54.5)  20 (65.5) 18 (90.0) 2 (10.0) | | 18 (54.5)  15 (45.5) 15 (100.0) 0 (0.0) | | 0.55 | |  |  |  |  |
| Time to stoma closure, months, median (IQR) | | | | | | 3.0 (4.0) | | 3.0 (2.0) | 0.59 | | | 3.0 (4.0) | | 3.0 (2.0) | | 0.79 | |  |  |  |  |
| Stoma closure, n (%)  Within three months  After three months | | | | | | 21 (54.3)  7 (33.30)  14 (66.70) | | 17 (42.5)  7 (41.20)  10 (58.80) | 0.63 | | | 20 (45.5) 7 (35.0) 13 (65.0) | | 15 (45.5) 6 (40.0) 9 (60.0) | | 0.77 | |  |  |  |  |
| Type of anastomosis, n (%)  Side-to-end  End-to-end  Not reported adequately | | | | | | 37 (80.4)  9 (19.6)  0 (0.0) | | 29 (70.0)  11 (27.5)  1 (2.5) | 0.36 | | | 35 (79.5)  9 (20.5)  0 (0.0) | | 22 (66.7)  10 (30.3)  1 (3.0) | | 0.29 | |  |  |  |  |
| Anastomotic leakage, n (%) | | | | | | 1 (2.2) | | 3 (7.5) | 0.25 | | | 1 (2.3) | | 3 (9.1) | | 0.19 | |  |  |  |  |
| Obstetric history, n = females | | | | | | **n=16** | | **N=17** |  | | | **n=16** | | **n=14** | |  | |  |  |  |  |
| Number of women who delivered a child, n (%) | | | | | | 14 (87.5) | | 13 (76.0) | 0.81 | | | 14 (87.5) | | 11 (78.6) | | 0.53 | |  |  |  |  |
| Woman with only vaginal deliveries, n (%) | | | | | | 12 (92.3) | | 13 (100.0) | 0.33 | | | 12 (92.3) | | 11 (100.0) | | 0.37 | |  |  |  |  |
| Presence of vaginal tears or episiotomy procedures, n (%) | | | | | | 8 (66.7) | | 5 (38.5) | 0.17 | | | 8 (66.7) | | 5 (45.5) | | 0.33 | |  |  |  |  |

** number of patients that completed measurements at M4.
All for the per protocol group:
^1^ Control group: a history of surgery for fistula (1x), endoscopic mucosal resection (EMR, 1x), endoscopic submucosal dissection (ESD, 1x), Transanal Endoscopic Microsurgery (TEM)/wait and see (3x). PFR group: a history of surgery for fistula (1x), TEM (1x), unknown type of surgery 20 years ago (1x).
^2^ All synchronic metastasis that were treated by primary resection (1x, control group), ablation (1x, control group) or complete response after neoadjuvant treatment (1x, control group), all before LAR.  ^3^ At index surgery or constructed in the first postoperative period (i.e. due to complications).

**Supplementary 2 Wexner incontinence score**M2: baseline measurement. M3: primary endpoint. M4: one-year follow-up. IQR: interquartile range. 95% CI: 95% Confidence interval.

| **Outcomes** | **Timing of measurement** | | **Intention to treat** | | | **Per protocol population** | | |
| --- | --- | --- | --- | --- | --- | --- | --- | --- |
|  |  |  | **Control group**  **n=46**** | **Intervention group  n=40**** | **p value** | **Control group**  **n=44**** | **Intervention group  n=33**** | **p value** |
| Total Wexner score, median (IQR) | M2 | | 6.00 (12.00) | 11.50 (8.00) | - | 5.50 (12.00) | 11.00 (8.00) | - |
|  | M3 | | 5.00 (7.00) | 6.00 (10.00) | - | 5.00 (4.00) | 6.00 (9.00) | - |
|  | M4 | | 4.50 (7.00) | 5.00 (6.00) | - | 4.00 (6.50) | 5.00 (6.00) | - |
| Change scores (M4-M2) | Adjusted mean difference * | | -2.54 95% CI -3.54 to -1.54 | -3.33  95% CI -4.41 to -2.26 | .30 | -2.67  95% CI -3.66 to -1.68 | -3.35 95% CI -4.50 to -2.20 | .39 |
| Change scores (M4-M3), | Adjusted mean difference * | | -1.02 95% CI -1.99 to -.05 | -1.02 95% CI -2.07 to .02 | .999 | -1.22 95% CI -2.18 to -.27 | -.89 95% CI -2.00 to .22 | .66 |
|  | | | **Control group** | **Intervention group** | **p value** |  | | |
| **At least moderate incontinence** | | | **n=27**** | **n=32**** | **.** |  |  |  |
| Total Wexner score, median (IQR) | M2 | | 12.00 (9.00) | 12.50 (7.00) | - |  |  |  |
|  | M3 | | 6.00 (8.00) | 7.50 (8.00) | - |  |  |  |
|  | M4 | | 7.00 (7.00) | 6.50 (6.75) | - |  |  |  |
| Change scores (M4-M2) | Adjusted mean difference * | | -4.23 95% CI -5.69 to -2.77 | -4.93 95% CI -6.27 to -3.59 | .49 |  |  |  |
| Change scores (M4-M3) | Adjusted mean difference * | | -1.40 95% CI -2.81 to .02 | -1.42 95% CI -2.71 to -.12 | .98 |  |  |  |
|  | | | **Control group** | **Intervention group** | **p value** |  |  |  |
| **No near-complete incontinence** | | | **n=40**** | **n=32**** | **.** |  |  |  |
| Total Wexner score, median (IQR) | M2 | | 5.00 (9.00) | 9.50 (8.00) | - |  |  |  |
|  | M3 | | 5.00 (4.00) | 5.00 (6.00) | - |  |  |  |
|  | M4 | | 4.00 (6.50) | 4.00 (5.50) | - |  |  |  |
| Change scores (M4-M2) | Adjusted mean difference * | | -1.43  95% CI -2.36 to -.50 | -2.81  95% CI -3.86 to -1.76 | .06 |  |  |  |
| Change scores (M4-M3) | Adjusted mean difference * | | -.55 95% CI -1.45 to .34 | -.75 95% CI -1.76 to .27 | .79 |  |  |  |
| **Between moderate and near-complete incontinence** | | | **n=21** | **n=24** | **p value** |  | | |
| Total Wexner score, median (IQR) | | M2 | 11.00 (8) | 11.50 (6) | - |  |  |  |
|  |  | M3 | 6.00 (6) | 6.50 (6) | - |  |  |  |
|  |  | M4 | 6.00 (5.5) | 5.50 (5.75) | - |  |  |  |
| Change scores (M4-M2), Mean (95%-CI) | | Unadjusted mean difference | -2.90  (-4.22 till -1.57) | -4.51  (-5.75 till -3.27_ | .083 |  |  |  |
|  |  | Adjusted mean difference * | -2.96  (-4.22 till -1.70) | -4.74  (-6.03 till -3.45) | .065 |  |  |  |
| Change scores (M4-M3), Mean (95%-CI) | | Unadjusted mean difference | -.70  (-2.03 till .63) | -1.10  (-2.34 till .14) | .662 |  |  |  |
|  |  | Adjusted mean difference * | -.76  (-2.06 till .53) | -.85  (-2.18 till .48) | .930 |  |  |  |

* ANCOVA with mean change in Wexner incontinence score (M2-M3 and M3-M4) adjusted for age, preoperatively assessed tumor height from anal verge, neoadjuvant treatment, and Wexner baseline score.
** Number of participants whom completed the M4 measurements.

**Supplementary 3: Secondary outcome measures, including a per protocol population analysis (FIQL, LARS score, EORTC QLQ-CR29).**M2: baseline measurement. M3: primary endpoint. M4: one-year follow-up. IQR: interquartile range. 95-CI: 95%-Confidence Interval.

| Table 3a. Faecal Incontinence related quality of life (FIQL) | | | | | | | |
| --- | --- | --- | --- | --- | --- | --- | --- |
|  | **Measurement** | **Intention to treat** | | | **Per protocol** | | |
|  |  | **Control group** | **PFR group** | **p value** | **Control group** | **PFR group** | **p value** |
| Non-selected patients | | **n=46**** | **n=40**** | **.** | **n=44**** | **n=33**** | **.** |
| Lifestyle | | | | | | | |
| Total score, median (IQR) | M2 | 2.80 (2.10) | 2.10 (1.68) | - | 2.80 (2.05) | 2.00 (1.98) | - |
|  | M3 | 3.10 (2.60) | 3.05 (1.57) | - | 3.10 (2.60) | 3.40 (1.55) | - |
|  | M4 | 2.85 (3.63) | 3.25 (1.60) | - | 2.95 (3.68) | 3.10 (1.65) | - |
| Change score (M4-M2) | Adjusted mean difference * | -.13  95% CI -.53 to .27 | .51  95% CI .08 to .93 | .03 | -.11  95% CI -.54 to .31 | .45  95% CI -.05 to .94 | .09 |
| Change score (M4-M3) | Adjusted mean difference * | -.11 95% CI -.60 to .39 | .23 95% CI -.30 to .75 | .37 | -.09 95% CI -.60 to .43 | .08 95% CI -.52 to .68 | .67 |
| Coping and behaviour | | | | | | | |
| Total score, median (IQR) | M2 | 2.22 (1.67) | 1.89 (1.08) | - | 2.22 (1.72) | 1.78 (1.17) | - |
|  | M3 | 2.33 (2.11) | 2.44 (1.61) | - | 2.33 (2.11) | 2.56 (1.75) | - |
|  | M4 | 2.39 (2.17) | 2.67 (1.50) | - | 2.39 (2.28) | 2.56 (1.50) | - |
| Change score (M4-M2) | Adjusted mean difference * | -.24  95% CI -.54 to .07 | .40  95% CI .07 to .73 | .01 | -.23 95% CI -.56 to .09 | .35 95% CI -.02 to .73 | .02 |
| Change score (M4-M3) | Adjusted mean difference * | -.19 95% CI -.57 to .19 | .24 95% CI -.16 to .65 | .13 | -.19 95% CI -.58 to .21 | .05  95% CI -.41 to .50 | .46 |
| Depression and self-perception | | | | | | | |
| Total score, median (IQR) | M2 | 2.70 (1.48) | 3.03 (1.38) | - | 2.70 (1.68) | 2.99 (1.58) | - |
|  | M3 | 2.54 (2.14) | 3.29 (1.13) | - | 2.54 (2.30) | 3.42 (1.22) | - |
|  | M4 | 2.64 (2.40) | 3.37 (1.71) | - | 2.64 (2.42) | 3.34 (2.13) | - |
| Change score (M4-M2) | Adjusted mean difference * | -.32 95% CI -.65 to .01 | .14 95% CI -.22 to .49 | .06 | -.32  95% CI -.66 to .03 | .08 95% CI -.32 to .48 | .14 |
| Change score (M4-M3) | Adjusted mean difference * | -.12 95% CI -.50 to .25 | -.08 95% CI -.49 to .32 | .88 | -.12 95% CI -.49 to .26 | -.25 95% CI -.68 to .19 | .66 |
| Embarrassment | | | | | | | |
| Total score, median (IQR) | M2 | 2.67 (2.33) | 3.00 (1.67) | - | 2.67 (2.33) | 3.00 (1.67) | - |
|  | M3 | 2.33 (2.67) | 3.17 (1.92) | - | 2.33 (3.00) | 3.17 (1.92) | - |
|  | M4 | 2.17 (3.42) | 2.67 (2.25) | - | 2.17 (3.58) | 2.67 (2.33) | - |
| Change score (M4-M2) | Adjusted mean difference * | -.58 95% CI -.99 to -.17 | -.09  95% CI -.54 to .35 | .11 | -.56  95% CI -1.00 to -.13 | -.16 95% CI -.66 to .35 | .23 |
| Change score (M4-M3) | Adjusted mean difference * | -.18 95% CI -.68 to .33 | -.16 95% CI -.70 to .39 | .95 | -.19 95% CI -.70 to .32 | -.34 95% CI -.94 to .25 | .70 |
| At least moderate incontinence at baseline | | **N=27** | **n=32** | **.** | **n=25** | **n=27** | **.** |
| Lifestyle | | | | | | | |
| Total score, median (IQR) | M2 | 2.90 (2.00) | 2.05 (1.30) | - | 2.90 (2.00) | 2.00 (1.90) | - |
|  | M3 | 3.10 (2.40) | 3.05 (1.35) | - | 3.10 (2.45) | 3.40 (1.30) | - |
|  | M4 | 3.00 (2.70) | 3.40 (1.52) | - | 3.40 (2.50) | 3.40 (1.70) | - |
| Change score (M4-M2) | Adjusted mean difference * | .14  95% CI -.29 to .57 | .73  95% CI.34 to 1.13 | .048 | .18 95% CI -.29 to .64 | .71 95% CI .27 to 1.16 | .10 |
| Change score (M4-M3) | Adjusted mean difference * | .16 95% CI -.39 to .71 | .14 95% CI -.37 to .64 | .95 | .19 95% CI -.41 to .80 | .06 95% CI -.52 to .64 | .75 |
| Coping and behaviour | | | | | | | |
| Total score, median (IQR) | M2 | 2.22 (1.67) | 1.83 (.89) | - | 2.22 (1.61) | 1.67 (1.22) | - |
|  | M3 | 2.00 (1.89) | 2.44 (1.17) | - | 2.00 (1.89) | 2.56 (1.67) | - |
|  | M4 | 2.78 (2.11) | 2.67 (1.19) | - | 2.78 (2.11) | 2.67 (1.44) | - |
| Change score (M4-M2) | Adjusted mean difference * | .01 95% CI -.32 to .34 | .64 95% CI .33 to .95 | .01 | .02 95% CI -.35 to .38 | .68 95% CI .34 to 1.03 | .01 |
| Change score (M4-M3) | Adjusted mean difference * | .09 95% CI -.32 to .49 | .19 95% CI -.19 to .56 | .71 | .09 95% CI -.35 to .53 | .12 95% CI -.31 to .54 | .93 |
| Depression and self-perception | | | | | | | |
| Total score, median (IQR) | M2 | 2.91 (1.08) | 2.87 (1.38) | - | 2.91 (1.13) | 2.82 (1.40) | - |
|  | M3 | 2.34 (1.88) | 3.46 (1.10) | - | 2.34 (1.85) | 3.54 (1.14) | - |
|  | M4 | 2.65 (2.34) | 3.51 (1.53) | - | 2.65 (2.34) | 3.56 (1.49) | - |
| Change score (M4-M2) | Adjusted mean difference * | -.27 95% CI -.66 to .12 | .26 95% CI -.10 to .62 | .049 | -.29 95% CI -.71 to .14 | .26 95% CI -.14 to .67 | .07 |
| Change score (M4-M3) | Adjusted mean difference * | .01 95% CI -.47 to .48 | -.13 95% CI -.57 to .30 | .67 | .03 95% CI -.46 to .52 | -.20 95% CI -.67 to .27 | .49 |
| Embarrassment | | | | | | | |
| Total score, median (IQR) | M2 | 2.67 (1.33) | 3.00 (1.58) | - | 2.67 (1.50) | 3.00 (1.67) | - |
|  | M3 | 2.33 (2.33) | 3.17 (1.33) | - | 2.33 (2.83) | 3.33 (1.67) | - |
|  | M4 | 2.33 (2.00) | 2.83 (2.08) | - | 2.33 (2.83) | 2.67 (1.33) | - |
| Change score (M4-M2) | Adjusted mean difference * | -.48 95% CI -.97 to .01 | .06 95% CI -.38 to .51 | .10 | -.50 95% CI -1.03 to .03 | .05 95% CI -.46 to .56 | .14 |
| Change score (M4-M3) | Adjusted mean difference * | -.11 95% CI -.72 to .50 | -.16 95% CI -.71 to .40 | .90 | -.12 95% CI -.77 to .53 | -.25 95% CI -.87 to .38 | .78 |
| Baseline Wexner <16 (no near-complete incontinence) | | **n=40** | **n=32** | **.** | **n=38** | **n=26** | **.** |
| Lifestyle | | | | | | | |
| Total score, median (IQR) | M2 | 3.05 (1.90) | 2.15 (1.90) | - | 3.05 (1.92) | 2.00 (2.48) | - |
|  | M3 | 3.15 (3.38) | 3.25 (1.48) | - | 3.30 (3.75) | 3.40 (1.47) | - |
|  | M4 | 2.80 (3.68) | 3.40 (1.63) | - | 2.90 (3.78) | 3.35 (1.82) | - |
| Change score (M4-M2), mean (95-CI) | Adjusted mean difference * | -.26  95% CI -.70 to .19 | .51  95% CI .01 to 1.00 | .03 | -.25 95% CI -.72 to .23 | .40 95% CI -.17 to .98 | .09 |
| Change score (M4-M3), mean (95-CI) | Adjusted mean difference * | -.14 95% CI -.69 to .41 | .22  95% CI -.40 to .84 | .39 | -.13 95% CI -.71 to .45 | .03  95% CI -.67 to .73 | .73 |
| Coping and behaviour |  |  |  |  |  |  |  |
| Total score, median (IQR) | M2 | 2.67 (1.69) | 2.11 (1.19) | - | 2.78 (1.53) | 1.83 (1.39) | - |
|  | M3 | 2.39 (2.28) | 2.56 (1.58) | - | 2.39 (2.36) | 2.78 (1.72) | - |
|  | M4 | 2.39 (2.28) | 2.67 (1.44) | - | 2.39 (2.39) | 2.61 (1.78) | - |
| Change score (M4-M2) | Adjusted mean difference * | -.32 95% CI -.65 to .02 | .43 95% CI .06 to .80 | .00 | -.32 95% CI -.68 to .04 | .35 95% CI -.09 to .78 | .02 |
| Change score (M4-M3) | Adjusted mean difference * | -.21 95% CI -.64 to .22 | .21  95% CI -.27 to .69 | .21 | -.21 95% CI -.65 to .23 | -.05 95% CI -.59 to .49 | .66 |
| Depression and self-perception |  |  |  |  |  |  |  |
| Total score, median (IQR) | M2 | 2.91 (1.46) | 2.99 (1.64) | - | 2.91 (1.49) | 2.99 (1.88) | - |
|  | M3 | 2.64 (2.34) | 3.47 (1.49) | - | 2.64 (2.46) | 3.51 (1.36) | - |
|  | M4 | 2.70 (2.41) | 3.54 (2.00) | - | 2.70 (2.44) | 3.40 (2.31) | - |
| Change score (M4-M2) | Adjusted mean difference * | -.32  95% CI -.68 to .05 | .19 95% CI -.22 to .59 | .07 | -.32 95% CI -.71 to .06 | .09 95% CI -.38 to .56 | .18 |
| Change score (M4-M3) | Adjusted mean difference * | -.14  95% CI -.56 to .28 | -.06 95% CI -.53 to .40 | .81 | -.13 95% CI -.54 to .28 | -.29 95% CI -.79 to .21 | .62 |
| Embarrassment |  |  |  |  |  |  |  |
| Total score, median (IQR) | M2 | 2.67 (2.42) | 3.33 (1.58) | - | 2.67 (2.67) | 3.00 (1.50) | - |
|  | M3 | 2.33 (3.17) | 3.33 (1.58) | - | 2.33 (3.50) | 3.33 (1.42) | - |
|  | M4 | 2.17 (3.58) | 2.67 (2.58) | - | 2.17 (3.67) | 2.67 (2.33) | - |
| Change score (M4-M2) | Adjusted mean difference * | -.58 95% CI -1.04 to -.13 | -.06 95% CI -.57 to .44 | .13 | -.56 95% CI -1.04 to -.08 | -.16 95% CI -.74 to .43 | .29 |
| Change score (M4-M3) | Adjusted mean difference * | -.16 95% CI -.72 to .40 | -.21 95% CI -.84 to .41 | .91 | -.18 95% CI -.75 to .39 | -.44 95% CI 1.13 to .25 | .57 |
| At least moderate incontinence at baseline and baseline Wexner <16 (no near-complete incontinence) | | **n=21** | **n=24** | **.** | **n=19** | **n=20** | **.** |
| Lifestyle | | | | | | | |
| Total score, median (IQR) | M2 | 3.00 (1.95) | 2.15 (1.80) | - | 3.00 (1.90) | 2.10 (2.13) | - |
|  | M3 | 3.10 (3.00) | 3.40 (1.33) | - | 3.10 (3.80) | 3.50 (1.40) | - |
|  | M4 | 3.00 (2.85) | 3.50 (1.20) | - | 3.80 (3.00) | 3.50 (1.65) | - |
| Change score (M4-M2) | Adjusted mean difference * | .02  95% CI -.48 to .51 | .79 95% CI .33 to 1.25 | .03 | .05 95% CI -.51 to .60 | .71 95% CI .18 to 1.25 | .08 |
| Change score (M4-M3) | Adjusted mean difference * | .17 95% CI -.49 to .82 | .10 95% CI -51 to .71 | .88 | .21 95% CI -.52 to .95 | -.02 95% CI -.74 to .70 | .65 |
| Coping and behaviour |  |  |  |  |  |  |  |
| Total score, median (IQR) | M2 | 2.22 (1.61) | 1.94 (1.19) | - | 2.22 (1.56) | 1.83 (1.39) | - |
|  | M3 | 2.22 (2.06) | 2.61 (1.61) | - | 2.22 (2.22) | 2.78 (1.64) | - |
|  | M4 | 2.78 (2.11) | 2.78 (1.28) | - | 2.78 (2.11) | 2.94 (1.36) | - |
| Change score (M4-M2) | Adjusted mean difference * | -.04 95% CI -.41 to .33 | .73 95% CI .39 to 1.09 | .00 | -.04 95% CI -.46 to .37 | .76 95% CI .36 to 1.17 | .01 |
| Change score (M4-M3) | Adjusted mean difference * | .12 95% CI -.38 to .63 | .13 95% CI -.35 to .60 | .99 | .13 95% CI -.42 to .69 | .01 95% CI -.53 to .55 | .76 |
| Depression and self-perception |  |  |  |  |  |  |  |
| Total score, median (IQR) | M2 | 2.91 (1.25) | 3.07 (1.28) | - | 2.91 (2.97) | 3.29 (1.47) | - |
|  | M3 | 2.54 (1.90) | 3.58 (3.18) | - | 2.54 (1.83) | 3.63 (.67) | - |
|  | M4 | 2.80 (2.31) | 3.69 (1.27) | - | 2.80 (2.29) | 3.74 (1.27) | - |
| Change score (M4-M2), mean (95-CI) | Adjusted mean difference * | -.26 95% CI -.72 to .20 | .37 95% CI -.06 to .79 | .05 | -.28 95% CI -.79 to .22 | .34 95% CI -.16 to .83 | .08 |
| Change score (M4-M3), mean (95-CI) | Adjusted mean difference * | .02 95% CI -.54 to .57 | -.12 95% CI -.64 to .40 | .71 | .05 95% CI -.53 to .63 | -.25 95% CI -.81 to .31 | .45 |
| Embarrassment |  |  |  |  |  |  |  |
| Total score, median (IQR) | M2 | 2.67 (1.50) | 3.33 (1.58) | - | 2.67 (1.67) | 3.17 (1.67) | - |
|  | M3 | 2.33 (3.00) | 3.33 (1.25) | - | 2.33 (3.33) | 3.33 (1.25) | - |
|  | M4 | 2.33 (2.83) | 3.33 (1.33) | - | 3.33 (1.25) | 2.33 (3.67) | - |
| Change score (M4-M2) | Adjusted mean difference * | -.46  95% CI -1.01 to .10 | .15 95% CI -.37 to .67 | .11 | -.47  95% CI -1.08 to .15 | .13 95% CI -.47 to .73 | .17 |
| Change score (M4-M3) | Adjusted mean difference * | -.06 95% CI -.78 to .66 | -.24 95% CI -.91 to .44 | .73 | -.08 95% CI -.86 to .70 | -.34 95% CI -1.10 to .42 | .63 |
| * ANCOVA with mean change in Wexner incontinence score (M2-M3 and M3-M4) adjusted for the FIQL baseline score Due to rounding, a difference in the mean scores and mean change scores may be possible. ** Number of participants whom completed the M4 measurements. | | | | | | | |

| Table 3b. Secondary outcome LARS score | | | | | | | |
| --- | --- | --- | --- | --- | --- | --- | --- |
|  | **Measurement** | **Intention to treat** | | | **Per protocol** | | |
|  |  | **Control group**  **n=46**** | **PFR group  n=40**** | **p value** | **Control group**  **n=44**** | **PFR group  n=33**** | **p value** |
| LARS score |  |  |  |  |  |  |  |
| Total LARS score, median (IQR) | M2 | 31.0 0(14.00) | 36.00 (8.00) | - | 29.00(13.00) | 36.00(9.00) | - |
|  | M3 | 31.0 0(16.00) | 31.00 (9.00) | - | 29.00 (16.00) | 31.00 (9.00) | - |
|  | M4 | 29.00 (13.00) | 31.00 (11.00) | - | 29.00 (14.00) | 30.00 (11.00) | - |
| Change score (M4-M2) | Adjusted mean difference * | -3.06 95% CI -5.25 to -.87 | -2.93 95% CI -5.29 to -.58 | .94 | -2.78 95% CI -5.08 to -.47 | -3.15 95% CI -5.83 to -.47 | .84 |
| Change score (M4-M3) | Adjusted mean difference * | -.76 95% CI -3.20 to 1.69 | -.36 95% CI -2.98 to 2.27 | .83 | -.44 95% CI -2.97 to 2.09 | -.08 95% CI -3.01 to 2.86 | .86 |
| LARS categories, n (%) | | | | | | | |
| No LARS  Minor LARS  Major LARS | M2 | 11 (21.6)  14 (27.5)  26 (51.0) | 4 (9.1)  5 (11.4)  35 (79.5) | .02 | 11 (22.4)  14 (28.6)  24 (49.0) | 3 (8.3)  4 (11.1)  28 (80.6) | .01 |
| No/minor vs. major LARS | M2 | - | - | <.01 |  |  | <.01 |
| No LARS  Minor LARS  Major LARS | M3 | 18 (35.3)  7 (13.7)  26 (51.0) | 7 (15.9)  11 (25.0) 26 (59.1) | .07 | 18 (36.7)  7 (14.3)  24 (49.0) | 6 (16.7)  9 (25.0)  21 (58.3) | .10 |
| No/minor vs. major LARS | M3 | - | - | .43 | - | - | .39 |
| No LARS  Minor LARS  Major LARS | M4 | 12 (26.10)  15 (32.60)  19 (41.30) | 7 (17.50)  8 (20.00)  25 (62.50) | .09 | 9 (20.50)  11 (25.00)  24 (54.50) | 3 (9.10)  4 (12.20)  26 (78.80) | .13 |
| No/minor vs. major LARS | M4 | - | - | .05 | - | - | .09 |
| * ANCOVA with mean change in Wexner incontinence score adjusted for the LARS baseline score Due to rounding, a difference in the mean scores and mean change scores may be possible. ** Number of participants whom completed the M4 measurements. | | | | | | | |

| Table 3c. Secondary measurement outcomes of EORT QLQ-CR29 | | | | | | | | |
| --- | --- | --- | --- | --- | --- | --- | --- | --- |
|  | | **Measurement** | **Intention to treat** | | | **Per protocol** | | |
|  |  |  | **Control group** | **PFR group** | **p value** | **Control group** | **PFR group** | **p value** |
| EORTC QLQ-CR29 | | | **n=45**** | **n=40**** |  | **N=43**** | **N=33**** |  |
| Body image | Mean (SD) | M2 | 14.0 (19.29) | 11.4 (14.7) | - | 12.7 (18.6) | 11.4 (15.1) | - |
|  |  | M3 | 14.2 (22.9) | 9.3 (13.3) | - | 12.9 (22.6) | 9.3 (11.7) | - |
|  |  | M4 | 12.6 (20.5) | 7.5 (13.4) | - | 10.85 (17.23) | 6.40 (11.46) | - |
|  | Change score (M4-M2), mean (95-CI) | Adjusted mean difference * | -2.02 -5.59 to 1.56 | -4.68 -8.47 to -0.89 | .31 | -2.49 -5.46 to 0.47 | -5.50  -8.89 to -2.12 | .19 |
|  | Change score (M4-M3), mean (95-CI) | Adjusted mean difference * | -1.61 -6.00 to 2.79 | -1.53  “-6.19 to 3.13 | .98 | -2.20 -6.56 to 2.15 | -2.18 -7.15 to 2.79 | .99 |
| Anxiety | | M2 | 26.7 (23.3) | 17.4 (22.1) | - | 25.7 (23.1) | 19.4 (23.1) | - |
|  |  | M3 | 24.7 (29.2) | 12.9 (16.4) | - | 23.8 (29.7) | 13.0 (16.5) | - |
|  |  | M4 | 21.5 (24.8) | 19.2 (22.5) | - | 20.16 (24.28) | 18.18 (18.80) | - |
|  | Change score (M4-M2), mean (95-CI) | Adjusted mean difference * | -1.17  -7.91 to 5.58 | -0.36  -7.52 to 6.81 | 0.87 | -2.36 -8.60 to 3.88 | -1.97 -9.10 to 5.15 | .94 |
|  | Change score (M4-M3), mean (95-CI) | Adjusted mean difference * | 1.18 -6.37 to 8.73 | 6.17 -1.85 to 14.19 | .37 | 0.19 -7.03 to 7.40 | 5.82 -2.43 to 14.06 | .31 |
| Weight | | M2 | 8.7 (16.2) | 13.6 (23.1) | - | 7.6 (15.7) | 13.0 (22.9) | - |
|  |  | M3 | 16.0 (22.6) | 12.9 (23.0) | - | 15.0 (22.6) | 13.0 (22.9) | - |
|  |  | M4 | 13.3 (19.3) | 13.3 (23.6) | - | 13.18 (19.44) | 14.14 (25.04) | - |
|  | Change score (M4-M2), mean (95-CI) | Adjusted mean difference * | 2.88  -2.35 to 8.10 | 0.10 -5.45 to 5.64 | 0.47 | 4.04 -1.14 to 9.32 | 1.80 -4.23 to 7.84 | .58 |
|  | Change score (M4-M3), mean (95-CI) | Adjusted mean difference * | -1.40 -5.90 to 3.09 | 0.74 -4.02 to 5.51 | .52 | -0.66 -5.23 to 3.91 | 1.87 -3.35 to 7.10 | .47 |
| Urinary Frequency | | M2 | 37.3 (29.8) | 40.9 (29.5) | - | 37.5 (30.5) | 40.7 (28.9) | - |
|  |  | M3 | 34.0 (34.0) | 30.3 (32.0) | - | 33.3 (34.0) | 28.7 (32.0) | - |
|  |  | M4 | 37.0 (33.5) | 34.2 (27.7) | - | 37.21 (33.50) | 35.35 (28.79) | - |
|  | Change score (M4-M2), mean (95-CI) | Adjusted mean difference * | -0.88 -8.91 to 7.15 | -5.68  -14.20 to 2.85 | .42 | -0.83 -9.09 to 7.42 | -4.98 -14.40 to 4.45 | .51 |
|  | Change score (M4-M3), mean (95-CI) | Adjusted mean difference * | 1.36 -7.96 to 10.68 | 5.14 -4.75 to 15.02 | .58 | 2.29 -7.07 to 11.66 | 7.11 -3.59 to 17.81 | .50 |
| Blood and Mucus in Stool | | M2 | 5.7 (11.0) | 8.3 (11.1) | - | 5.6 (11.1) | 6.9 (8.3) | - |
|  |  | M3 | 4.7 (8.9) | 5.3 (11.8) | - | 4.8 (9.0) | 4.6 (10.2) | - |
|  |  | M4 | 6.7 (16.8) | 5.0 (9.4) | - | 6.98 (17.15) | 4.55 (8.61) | - |
|  | Change score (M4-M2), mean (95-CI) | Adjusted mean difference * | -0.08 -4.11 to 3.95 | -2.41 -6.69 to 1.87 | .43 | 0.73 -3.54 to 5.00 | -1.97 -6.84 to 2.91 | .41 |
|  | Change score (M4-M3), mean (95-CI) | Adjusted mean difference * | 2.01 -2.08 to 6.10 | 0.24 -4.10 to 4.58 | .56 | 2.22 -2.19 to 6.62 | 0.65 -4.38 to 5.67 | .64 |
| Stool frequency | | M2 | 35.3 (19.8) | 40.2 (20.7) | - | 34.7 (19.7) | 36.6 (20.2) | - |
|  |  | M3 | 27.0 (23.1) | 31.1 (20.2) | - | 25.2 (22.1) | 27.8 (17.4) | - |
|  |  | M4 | 26.3 (20.3) | 29.2 (21.9) | - | 25.97 (20.67) | 25.25 (20.04) | - |
|  | Change score (M4-M2), mean (95-CI) | Adjusted mean difference * | -11.40 -17.00 to -5.81 | -10.51 -16.44 to -4.58 | .83 | -10.40 -16.04 to -4.77 | -11.70 -18.13 to -5.26 | .76 |
|  | Change score (M4-M3), mean (95-CI) | Adjusted mean difference * | -0.92 -7.51 to 5.68 | -1.89 -8.88 to 5.11 | .84 | 0.03 -6.52 to 6.58 | -2.56 -10.04 to 4.92 | .61 |
| Urinary incontinence | | M2 | 6.7 (15.1) | 9.9 (17.0) | - | 6.9 (15.3) | 9.3 (15.1) | - |
|  |  | M3 | 7.3 (14.5) | 6.8 (13.6) | - | 7.5 (15.6) | 7.4 (14.1) | - |
|  |  | M4 | 5.9 (14.7) | 12.5 (18.0) | - | 6.20 (15.01) | 13.13 (18.52) | - |
|  | Change score (M4-M2), mean (95-CI) | Adjusted mean difference * | -0.80 -4.82 to 3.23 | 3.40 -0.87 to 7.67 | .16 | -0.42 -4.51 to 3.66 | 4.59  -0.07 to 9.25 | .11 |
|  | Change score (M4-M3), mean (95-CI) | Adjusted mean difference * | -1.43 -5.38 to 2.53 | 4.94 0.75 to 9.13 | .03 | -0.71 -4.68 to 3.27 | 4.96 0.43 to 9.50 | .07 |
| Dysuria | | M2 | 2.0 (10.5) | 4.6 (11.6) | - | 2.1 (10.7) | 4.6 (11.7) | - |
|  |  | M3 | 1.3 (6.6) | 0.8 (5.0) | - | 1.4 (6.7) | 0.0 (0.0) | - |
|  |  | M4 | 0.7 (5.0) | 1.7 (7.4) | - | 0.78 (5.08) | 1.01 (5.80) | - |
|  | Change score (M4-M2), mean (95-CI) | Adjusted mean difference * | -2.33 -4.17 to -0.49 | -1.55  -3.50 to 0.40 | .57 | -2.31 -3.97 to -0.66 | -2.04  -3.93 to -0.15 | .83 |
|  | Change score (M4-M3), mean (95-CI) | Adjusted mean difference * | -1.05 -2.92 to 0.83 | 1.18 -0.81 to 3.16 | .11 | -.105 -3.04 to 0.95 | 1.37 -0.91 to 3.65 | .12 |
| Abdominal Pain | | M2 | 14.7 (21.5) | 14.4 (25.3) | - | 14.6 (21.6) | 16.7 (27.0) | - |
|  |  | M3 | 9.3 (19.1) | 13.6 (25.2) | - | 9.5 (19.2) | 11.1 (21.1) | - |
|  |  | M4 | 7.4 (14.0) | 8.3 (18.1) | - | 6.98 (13.72) | 8.08 (18.69) | - |
|  | Change score (M4-M2), mean (95-CI) | Adjusted mean difference * | -7.37 -11.83 to -2.91 | -5.88  -10.61 to -1.15 | .65 | -8.42 -12.85 to -3.99 | -7.21 -12.27 to -2.16 | .72 |
|  | Change score (M4-M3), mean (95-CI) | Adjusted mean difference * | -2.80 -9.92 to 4.31 | -6.02  -13.56 to 1.53 | .54 | -3.85 -10.10 to 2.40 | -3.07  -10.20 to 4.07 | .87 |
| Buttock Pain | | M2 | 24.0 (29.4) | 23.5 (31.8) | - | 22.2 (27.8) | 23.1 (32.7) | - |
|  |  | M3 | 15.3 (22.5) | 12.1 (25.0) | - | 15.0 (22.6) | 11.1 (22.5) | - |
|  |  | M4 | 17.0 (25.2) | 13.3 (27.0) | - | 14.73 (22.19) | 10.10 (19.52) | - |
|  | Change score (M4-M2), mean (95-CI) | Adjusted mean difference * | -7.59 -14.84 to -0.34 | -11.46  -19.15 to -3.78 | .47 | -8.41 -14.72 to -2.10 | -13.29 -20.49 to -6.09 | .31 |
|  | Change score (M4-M3), mean (95-CI) | Adjusted mean difference * | -0.03 -8.98 to 8.93 | 0.03 -9.47 to 9.53 | .99 | -2.52 -10.80 to 5.77 | -1.78  -11.23 to 7.69 | .91 |
| Bloating | | M2 | 16.0 (23.6) | 17.4 (23.3) | - | 15.3 (23.8) | 16.7 (23.2) | - |
|  |  | M3 | 18.0 (22.5) | 15.9 (22.1) | - | 16.3 (21.6) | 13.0 (16.5) | - |
|  |  | M4 | 11.9 (19.0) | 15.8 (20.0) | - | 10.85 (18.86) | 15.15 (20.57) | - |
|  | Change score (M4-M2), mean (95-CI) | Adjusted mean difference * | -5.64 -10.59 to -0.69 | -1.15 -6.40 to 4.10 | .22 | -5.78 -10.79 to -0.77 | -0.55  -6.27 to 5.17 | .18 |
|  | Change score (M4-M3), mean (95-CI) | Adjusted mean difference * | -5.22 -12.28 to 1.83 | 0.04 -7.44 to 7.52 | .31 | -4.79 -11.39 to 1.80 | 3.22 -4.31 to 10.75 | .12 |
| Dry Mouth | | M2 | 12.0 (17.5) | 15.2 (20.9) | - | 11.1 (17.3) | 12.0 (16.2) | - |
|  |  | M3 | 17.3 (24.5) | 15.2 (23.2) | - | 17.7 (24.6) | 13.9 (21.6) | - |
|  |  | M4 | 15.6 (22.0) | 14.2 (22.5) | - | 14.73 (22.19) | 12.12 (18.29) | - |
|  | Change score (M4-M2), mean (95-CI) | Adjusted mean difference * | 4.26 -1.39 to 9.91 | -0.63 -6.62 to 5.37 | .24 | 4.90 -0.78to 10.59 | 0.68 -5.81 to 7.17 | .33 |
|  | Change score (M4-M3), mean (95-CI) | Adjusted mean difference * | 0.00  -5.60 to 5.60 | 0.00  -5.94 to 5.94 | .60 | -0.83 -6.52 to 4.86 | 0.07  -6.43 to 6.57 | .84 |
| Hair Loss | | M2 | 2.0 (10.5) | 1.5 (10.1) | - | 2.1 (10.7) | 1.9 (11.1) | - |
|  |  | M3 | 0.7 (4.7) | 1.5 (7.0) | - | 0.7 (4.8) | 1.9 (7.7) | - |
|  |  | M4 | 0.7 (5.0) | 0.0 (0.0) | - | 0.78 (5.08) | 0.00 (0.00) | - |
|  | Change score (M4-M2), mean (95-CI) | Adjusted mean difference * | -0.83 -1.91 to 0.25 | -1.57  -2.71 to -0.42 | .35 | -0.98 -2.15 to 0.19 | -1.75 -3.09 to -0.42 | .39 |
|  | Change score (M4-M3), mean (95-CI) | Adjusted mean difference * | 0.00 -1.88 to 1.88 | -0.83 -2.83 to 1.16 | .55 | 0.00 -2.04 to 2.05 | -1.01 -3.35 to 1.32 | .52 |
| Taste | | M2 | 1.3 (6.6) | 6.1 (14.9) | - | 0.7 (4.8) | 6.5 (15.6) | - |
|  |  | M3 | 0.0 (0.0) | 2.3 (8.5) | - | 0.0 (0.0) | 1.9 (7.7) | - |
|  |  | M4 | 2.2 (11.0) | 0.8 (5.3) | - | 0.78 (5.08) | 0000 | - |
|  | Change score (M4-M2), mean (95-CI) | Adjusted mean difference * | -0.08 -2.64 to 2.48 | -2.41 -5.13 to 0.31 | .22 | -1.42 -2.61 to -0.23 | -2.19 -3.56 to -0.83 | .40 |
|  | Change score (M4-M3), mean (95-CI) | Adjusted mean difference * | 2.70 0.41 to 5.00 | -0.54 -2.98 to 1.90 | .06 | .78 -0.41 to 1.96 | 0.00 -1.36 to 1.36 | .40 |
| Flatulence | | M2 | 40.0 (23.3) | 40.9 (25.8) | - | 39.6 (23.5) | 39.8 (23.7) | - |
|  |  | M3 | 37.3 (27.5) | 34.9 (24.9) | - | 35.4 (27.6) | 33.3 (26.4) | - |
|  |  | M4 | 34.1 (29.7) | 36.7 (24.8) | - | 32.56 (29.54) | 34.34 (22.80) | - |
|  | Change score (M4-M2), mean (95-CI) | Adjusted mean difference * | -7.70 -15.38 to -0.01 | -5.51 -13.66 to 2.64 | .70 | -8.36 -15.96 to -0.76 | -6.28 -14.95 to 2.39 | .72 |
|  | Change score (M4-M3), mean (95-CI) | Adjusted mean difference * | -4.51 -12.92 to 3.91 | 0.90 -8.02 to 9.83 | .38 | -4.62 -13.05 to 3.82 | -0.05 -9.68 to 9.58 | .48 |
| Faecal incontinence | | M2 | 19.3 (30.2) | 18.2 (22.1) | - | 18.1 (29.9) | 18.5 (21.7) | - |
|  |  | M3 | 14.7 (22.5) | 16.7 (26.4) | - | 13.6 (22.5) | 15.7 (24.5) | - |
|  |  | M4 | 12.6 (21.7) | 13.3 (22.4) | - | 10.86 (20.21) | 11.11 (15.96) | - |
|  | Change score (M4-M2), mean (95-CI) | Adjusted mean difference * | -7.51 -13.02 to -2.00 | -5.72 -11.56 to 0.12 | .66 | -8.21 -12.80 to -3.62 | -7.48 -12.72 to -2.24 | .84 |
|  | Change score (M4-M3), mean (95-CI) | Adjusted mean difference * | -2.94 -9.04 to 3.17 | -3.36 -9.84 to 3.11 | .92 | -3.84 -10.17 to 2.48 | -5.10 -12.31 to 2.12 | .80 |
| Sore Skin | | M2 | 6.7 (24.5) | 23.5 (31.0) | - | 16.0 (24.8) | 19.4 (28.0) | - |
|  |  | M3 | 10.7 (19.6) | 14.4 (23.2) | - | 9.5 (19.2) | 13.0 (20.0) | - |
|  |  | M4 | 16.3 (24.2) | 14.2 (27.1) | - | 14.73 (23.35) | 11.11 (19.84) | - |
|  | Change score (M4-M2), mean (95-CI) | Adjusted mean difference * | -2.16 -9.01 to 4.70 | -7.58 -14.85 to -0.30 | .29 | -2.55 -8.86 to 3.76 | -7.79  -15.00 to -0.58 | .28 |
|  | Change score (M4-M3), mean (95-CI) | Adjusted mean difference * | 4.69 -3.37 to 12.75 | -1.11 -9.66 to 7.44 | .33 | 3.69 -4.09 to 11.48 | -2.79 -11.69 to 6.10 | .28 |
| Embarrassment | | M2 | 24.7 (28.4) | 25.8 (26.8) | - | 23.6 (28.3) | 24.1 (26.0) | - |
|  |  | M3 | 15.3 (22.5) | 15.2 (23.2) | - | 14.3 (22.6) | 13.9 (21.6) | - |
|  |  | M4 | 11.9 (20.3) | 13.3 (19.7) | - | 11.63 (20.42) | 12.12 (18.29) | - |
|  | Change score (M4-M2), mean (95-CI) | Adjusted mean difference * | -15.08 -20.51 to -9.76 | -13.87 -19.52 to -8.22 | .76 | -13.86 -19.19 to -8.53 | -13.26 -19.34 to -7.17 | .88 |
|  | Change score (M4-M3), mean (95-CI) | Adjusted mean difference * | -3.03 -8.90 to 2.84 | -0.76  -6.99 to 5.47 | .60 | -2.30  -8.03 to 3.43 | -0.03  -6.57 to 6.51 | .60 |
| * ANCOVA with mean change in Wexner incontinence score (M2-M3 and M3-M4) adjusted for the EORTC QLQ-CR29 baseline score. Due to rounding, a difference in the mean scores and mean change scores may be possible. ** Number of participants whom completed the M4 measurements. | | | | | | | | |
